# A Complete Treatise on the Art of Midwifery; or, Theoretical and Practical Tocology, with a Compendium of Those Maladies Which Complicate Pregnancy, Labour, and the Puerperal Condition, and of Those to Which Newborn Infants Are Liable

**Published:** 1835-04-01

**Authors:** 


*Traité complet de l'Art des Accouchemens, &c.—A Complete Treatise on the Art of Midwifery; or, Theoretical and Practical Tocology, with a Compendium of those Maladies which complicate Pregnancy, Labour, and the Puerperal Condition, and of those to which Newborn Infants are liable. Sixteen Plates.* By Alfred Velpeau, Professor of Clinical Surgery to the Faculty of Medicine at Paris; Surgeon to the Hôpital de la Pitié, &c. *Second Edition, corrected and augmented.*—Paris and London, 1835. 8vo. Vol. I. pp. 572; Vol. II. pp. 580.

It is customary, and it is generally necessary for reviewers, so numerous are the works that are constantly issuing from the press, to dismiss a second edition with a brief survey of those chapters which have undergone considerable alteration; but we shall, upon the present occasion, deviate from the ordinary rule, and give to M. Velpeau's work as lengthened a consideration as if he had not before addressed the profession upon the same subject. The present treatise is indeed rather a new work, than a new edition. The first edition was merely an abstract of the author's lectures, and intended merely for students: that before us contains three times as much matter as its predecessor, and every important subject has been rewritten and remodelled.

We are so accustomed to meet with promises in prefaces that seem to be altogether lost sight of in the work itself, that we usually pass over the latter with indifference, and hardly expect that the former will be fulfilled. M. Velpeau assures us, in *his* preface, that he has endeavoured to be unjust to no man; that he has spoken of all without hatred or prejudice, but at the same time without enthusiasm, and with the most perfect independence. "The sciences form a republic, in which every man should be at liberty to search and examine; to have his opinions, and to state what he thinks. Truth is the avowed object of all those who cultivate them; but, as truth may be arrived at in various ways, I could never understand why a rational man should be offended because his ideas were not regarded as a law by others." We like the spirit with which this is said, and we like still more the fidelity with which we find, upon an attentive perusal,—not a hasty chapter-of-contents view of the book,—M. Velpeau has acted up to his prefatorial professions. Our opinion of the general character and merit of his work will be in better place after we have given a sketch of its contents; but we may tempt our readers to follow us more readily by at once giving to him full title to the claim of perfect impartiality in all his discussions. Tros, Tyrinsve,

French, German, or English writers, are all frankly, but upon the whole very fairly, treated. Still more too to his credit, he holds the balance as evenly with respect to his own opinions, and not unfrequently admits he may be mistaken, from respect to the opinions of others, even upon subjects to which he has devoted a large share of his attention. "*Peut-être je me trompe moi-même*," is, in our estimation, a phrase of good import: it justifies confidence where a decided tone is assumed.

The term *tocology*, derived from τοκος, labour, and λογος, study, which M. Velpeau would fain introduce for the more homely word Accouchement, or midwifery, now in general use in this country, is classically unobjectionable, but we doubt whether it will ever come into general use.

In a short introduction, M. Velpeau touches very properly upon a subject, which in this country also has been discussed with more of acrimony than justice, the importance of the art and science of midwifery. He briefly but completely refutes the opinions, or rather the assertions, of those who have vilified the practice, and attempted to degrade the practitioner of this branch of medical and surgical science. We have ourselves grown grey in the practice of midwifery, and feel, we trust, a laudable wish that our junior contemporaries may not be misled by a few cynical philosophers,—knights-errant against the science of midwifery, who, by means of letters in the "Times," and circulars "to the Ladies," have in vain exerted their little power to lead the public to believe that midwifery is an art which is at once "degrading to those who practise it, and utterly useless to the public." Roussel, in France, was one of the leading advocates of this doctrine, and we confess that the eloquence of *his* language might sometimes make us forget the weakness of his arguments. In this country, we have no such formidable foes to contend with; for here the talk of our contemners is only dangerous to the cause they plead. There was indeed a time when the duties of the accoucheur were limited to attendance during the act of labour, and to the administration of a few simples, or to the trial of charms, after it. Even then he was useful in his calling: but now he claims a higher rank in the profession; for not only is he looked to for assistance during the act of labour, but, by common consent, the public intrust to him many of the most serious maladies of women and children.

Among many causes which long contributed to check that improvement in the art of midwifery, which zeal and talent effected in other branches of medical science, none more effectually barred its progress than the popular, and even professional, belief of its inutility. But, without drawing any invidious comparison between the importance of the various duties that are now intrusted to the accoucheur and other medical practitioners, it may be fairly and honestly asserted, that they are not inferior to any which more especially belong to other branches of the healing art. It is therefore not only deceptive to the student, but dangerous and cruel to society at large, to conceal from him the responsibility he imposes upon himself in undertaking the duties of midwifery. He who expects to have no difficulties to contend with, will make no preparations to avert them; and, when he meets with perplexities, of which he had been taught to disbelieve the existence, he fancies that none were ever so entangled before him, and sinks speedily into despair. We hope and believe that the young men of the present day have too much common sense to be induced, by any wandering lecturer, to neglect the study of midwifery, from a belief that it offers no subjects worthy of their attention, or that the skilful practitioner of it has not the same and as frequent opportunities as others of his brethren of mitigating the sufferings of his patients, or of snatching them from danger. The most prominent feature, says Dr. Bisset Hawkins,* afforded by medical statistics, next to the diminished mortality of infancy, is the remarkable change which has supervened within the last century in the fate of lying-in women. In 1750, at the British Lying-in Hospital of London, one woman died out of forty-two. In 1780, one died in sixty; and, lastly, the improvement became so great, that only one case was fatal out of 288, in the ten years between 1789 and 1798. The deaths of the children during all this period preserved a constant proportion to the fate of the mothers. In 1758, one newborn child died out of fifteen; in 1780, one in forty-four; and in the last decade, from 1789 to 1798, only one in seventy-seven. Various other reports, from public and private sources, might be adduced of a similar kind, to establish the fact of the rapid decrease in the mortality of lying-in women and children; and, as an anonymous writer cannot be accused of egotism, the reviewer may safely state that, in twenty-three years' active practice, during which time he has attended upwards of two thousand women during and after labour, but four have died of disease connected with parturition, and not one during or within a week after their confinement. To what cause can such a decrease

* Elements of Medical Statistics. By F. Bisset Hawkins, M.D. 1829.—Rev.

of mortality be owing, but to the increased skill of practitioners of midwifery; and what better proof can be given of the utility of their art?

We pass over M. Velpeau's historical notice of the progress of midwifery, not because it is without interest, but that we may enter upon more practical matter. It is immediately followed by a synopsis of the divisions of labour, adopted by different French and German writers; and statistical records of the mortality of lying-in women, in different public establishments for their reception.

The first 110 pages of the work are occupied with a good account of the pelvis and female sexual organs. Important as these subjects are to the student, they offer but little matter for comment to the reviewer. They comprise indeed the grammar of midwifery, and, without a thorough knowledge of them, no man can practise, with safety to his patients, or with that degree of self-confidence which is essential to his own comfort. Hence, then, we cannot but regret that some of our midwifery lecturers omit these subjects altogether, or treat them too superficially, and trust them to the anatomical teacher, who gives little if any time to those points which claim the greatest attention from the obstetrical student.

At page 15 we see an error of the press, which might perplex a tyro. In describing the axis of the *superior* strait of the pelvis, the word *inferior* is printed instead of superior.

There is one point of practical importance connected with the subject of deformity of the pelvis, to which we think M. Velpeau too briefly alludes. With the fact to which we refer the practitioner should be acquainted, that he may neither injure his own reputation by a hasty opinion, or create unnecessary alarm in the mind of his patient. He must not infer that the pelvis is deformed, because there is distortion of the spine. We know, from our own experience, as well as from the testimony of others, that *great* curvature of the spine may exist, and yet the woman have very easy labours. On the other hand, the pelvis may be distorted where the spine is not. The pelvis will seldom be found affected, unless distortion of the spine began in early childhood. When the curvature begins after the age of twelve, the pelvis seldom suffers; but, if the distortion appeared before the age of five, the pelvis is probably narrow. The late Mr. John Shaw remarks,* "that, in whatever state of distortion the spine and ribs may be, the bones of the pelvis

* On Spinal Distortion.

will not be found distorted, unless there be at the same time marks of rickets in some of the long or solid bones." Wilson, Ward, Stafford, and Beale, in their treatises on Spinal Distortion, confirm this opinion. We may mention a curious case, in illustration of the existence of great spinal distortion without pelvic deformity. We forget our authority, but we made a note of it immediately after reading an account of it.

A woman, aged forty, presented herself at one of the hospitals in Paris, to be treated for some chronic affection. Besides the most whimsical distortion of her spine, her chest was depressed, and she had a considerable protuberance on her back. She said she had become deformed at the age of eighteen, in consequence of some severe disease. She informed M. Dubois that she had had six children, and that she had never suffered the slightest difficulty in any of her labours.

Now, in such and similar instances, unless the practitioner were acquainted with the fact, he might not only give an erroneous opinion, and unnecessarily prevent a crooked lady from straightway being married, but he might also have recourse to hazardous and unnecessary operations in time of labour, from the conviction that the pelvis must be distorted because there was great general deformity.

M. Velpeau next treats on the physiology of Menstruation, and gives a good and detailed account of the subject. The immediate source of the menstrual discharge has been long a disputed point. Some, with Vesalius,* have presumed it flowed from the veins; others, with Ruysch,† from the arteries, or from the capillaries, with Winslow and Meibomius. M. V. believes "that the menstrual discharge is a secretion from the uterus, exhaling or perspiring from the organ, but that it is not known whether it transudes rather from the venous than the arterial capillaries." In our opinion, he prudently abstains from wasting time in discussing the often disputed, but never to be determined, question of the final causes of menstruation, or why the discharge occurs periodically. Cabanis‡ truly says, that the philosophy of final causes has never yet been able to bear the test of a rigid scrutiny, although the vain yet limited mind of man has induced him to reject the mortifying truth: and, with respect to the periodicity of the menstrual efflux, all we can say is, that all the periodical phenomena of health and disease have hitherto appeared among the inexplicable mysteries of animal

* De Fabrica Corp. Human. lib. v. cap. 15.
† Epist. ad Boerhaave, &c.
‡ Du Degré de Certitude de la Medecine.—Rev.

nature. We know not why the menses should be thrown off once a month, rather than after intervals of any other duration. M. Velpeau briefly mentions two cases, which merit the attention of many practitioners, who are too much in the habit of indulging the popular belief that, if the menses do not regularly flow in a female arrived at the age of puberty, medical means should be had recourse to, for the purpose of soliciting or of forcing the discharge. Now we take it, that, provided the health of the female does not suffer, the mere absence of the menses does not justify our interference. "I know," says the author, "a young lady, aged twenty-five, who never menstruated from the age of eighteen, and who still enjoyed perfect health." Again, a lady, æt. thirty-two, had never been "unwell" since the age of twenty-one: she also remained in good health. We know, from ample experience, that great injury is frequently inflicted upon young females, by the pertinacious use of forcing or emmenagogue remedies, as they are termed, merely because the age of puberty has arrived, and the menses do not appear. But girls are very often "backward;" they may be women in age, but children in form, retaining all the characteristic traits of childhood, and having none of the ordinary indications of approaching womanhood. Here nature has not prepared the constitution for the performance of the menstrual function, and the too common practice of endeavouring to hasten it by artificial means is injudicious and dangerous. We can readily excuse popular prejudices upon the subject; but the medical practitioner can know but little of the limits to which his art should be confined, if he endeavours to drive nature to establish a function for which she is yet unprepared.

With respect to the cessation of the menses, (that much-dreaded period of female life, when most women suffer from fancy, more than reality,) it should be borne in mind that, from the extensive statistical researches of MM. Châteauneuf and Lachaise, as many men die as women between the ages of forty and forty-five. We do not deny that, at this "critical" age of female life, the health does not sometimes suffer, and that disease, which had been previously lurking in the system, is now occasionally aggravated; but we *do* affirm, that the popular belief, and the professional doctrine, which attach so many evils to the cessation of the menses, are greatly exaggerated. The age is "critical" indeed to every woman; but it is more so because her beauty fades, than because her health declines.

The next subject we approach is Reproduction, the most wonderful phenomenon of animated nature, and one which has naturally excited the deepest attention of philosophers. As we do not believe our readers would thank us for occupying our pages with the fable of bygone days, we pass over the sketch M. Velpeau has given of the thousand-and-one hypotheses that have at various periods been hazarded upon this subject.

Upon the much-disputed points connected with the formation, and even the appearance, of the corpus luteum, our author remains in doubt, and thinks, as we do, that it is a question "qui exige evidemment de nouvelles recherches." He has not referred to one, and in our opinion the best, authority upon this very interesting and really important topic: we mean to a paper by Dr. Montgomery, "on the Signs of Pregnancy and Delivery," in the 17th and 18th parts of the Cyclopædia of Practical Medicine. Dr. M. sums up a very careful examination of the subject by affirming his conviction of the truth of the two propositions of Haller, viz. that "conception never happens without the production of a corpus luteum," and that "the corpus luteum is never found in virgin animals, but is the effect of impregnation;" and he thinks that those who have supposed or asserted that the corpora lutea may exist without impregnation, and of course be found in the virgin ovary, have been led into the error by confounding appearances and structures essentially different, and in fact having only one character in common, which is their colour; altogether forgetting that "every yellow substance in the ovary is not a corpus luteum." When Mr. Angus was tried at Liverpool, in 1808, for the supposed murder of Miss Burns,* great doubt arose as to whether the condition of the uterus, or its appendages, was such as to prove a pregnancy recently existing. "It was not until after the trial that the ovaria were examined: they were then divided in the presence of a number of physicians, and a corpus luteum distinctly perceived in one of them." Mr. Hay took the uterus and its appendages to London, and shewed it to the most eminent practitioners: he received certificates from Drs. Denman and Haighton, Messrs. Henry Cline, C. M. Clarke, Astley Cooper, and Abernethy, all stating that it exhibited appearances that could *alone* be explained on the idea of an advanced state of pregnancy; *and it appears to have been universally allowed that the discovery of the corpus luteum proved the fact beyond a doubt.*

* Dr. Montgomery's paper, *loc. cit.*, from the report of the trial.—Ed. Med. and Surg. Journal, vol. v. p. 220.

In the chapter on "Vraie Grossesse," (true pregnancy, in contradistinction to false or apparent pregnancy,) M. Velpeau gives a very detailed and accurate account of the anatomical changes that occur during utero-gestation. He believes, with Desormeaux, that the cervix uteri loses about one third of its length at the fifth month, one half at the sixth, two thirds or three fourths in the seventh, and that the remainder is effaced during the ninth. We are guarded, however, and very properly too, from taking these rules as infallible guides. "Repeated observation, and the most carefully conducted experiments, have singularly lessened the confidence which I once was inclined to grant them: the changes which the cervix uteri undergoes during pregnancy vary almost as much as its anatomical characters in the unimpregnated female."

In determining the question of pregnancy, or the period, we are often at least confined to presumptive evidence, and, as such, we would not reject the condition and progressive changes of the cervix uteri; but abundant experience convinces us that the dogmatic rules laid down in books upon this subject are calculated to deceive the practitioner, even supposing that he has the tact to distinguish the progressive changes of the cervix. Dr. Gooch says,* "the examination of many uteri has taught me that the neck of the uterus is as much altered in some women at the fourth month as in others at the sixth, especially in those who have had several children, in whom the neck yields more readily than in the first pregnancies." "The neck of the womb," says Smellie,† "will in some be felt as long in the eighth, as in others in the sixth or seventh, month. This variation sometimes makes the examination of the abdomen more certain than the touch of the vagina, and so *vice versâ*. At other times we must judge by both."

The thickness of the uterine parietes during pregnancy is a subject which has been much contested by obstetricians. M. Velpeau says, that the uterine parietes retain nearly the same thickness during the whole course of utero-gestation as in the unimpregnated state. It varies, however, greatly in different women, and at different parts of the organ. We have now before us eight unimpregnated and healthy uteri, taken from young females, all of them differing greatly in substance as well as form. Their substance varies from one half to nearly three quarters of an inch. In one, the parietes are not above three lines in substance. Our friend, Mr.

* Account of some of the most important Diseases of Women, p. 214.—Rev.
† Treatise on Midwifery, Vol. i. p. 188. Fifth Edition.—Rev.

Griffith, has a uterus in his possession, so thin as to be nearly transparent. In three gravid uteri, at the full period, also in our view, we perceive the same difference in the thickness of the parietes; but generally the parietes of the uterus at the ninth month are decidedly thinner than in the unimpregnated state. Those who have asserted that the womb is much thickened during pregnancy, have probably been misled by examining the organ a few days after labour, when the parietes, as we now perceive, in preparations before us, are really thickened from contraction. In one of these preparations, a section of a uterus we removed from a woman twenty-four hours after labour, the parietes are full an inch thick; and often the substance is greater, especially at the fundus.

To explain the extraordinary development of the cavity of the uterus during pregnancy, the ancient writers, and even Mauriceau,* supposed that the ovum, as it increased in size, dilated the uterus. It is certain, however, that the dilating power resides in the uterus itself, and that it is quite independent of the mechanical distension produced by the product of conception. In extra-uterine pregnancy, for example, (as we see in a valuable preparation before us,) the uterus dilates as in ordinary gestation.

"To explain the dilatation of the pregnant uterus, it is useless to imagine, with Malpighi, a principle of fermentation contained in the semen, or, with Blumenbach, a particular vital action. The turgescence of the uterus produced by impregnation, and kept up by the ovum, satisfactorily explains the fact. The congestion of the uterus produces in the organ an excess of nutrition. The new molecules that are deposited in it necessarily elongate its fibres. The vascular canals are lengthened and enlarged at the same time. This elongation cannot take place without augmenting the extent of the circles, or the curves, which each fibre and each vessel of the organ represents; and thus the developement of its cavity is an inevitable consequence of the augmented nutrition of its parietes." (P. 168.)

During pregnancy, the bladder mounts above the brim of the pelvis; the urethra is hidden behind the symphysis pubis, and has nearly a vertical direction; the meatus is deep within the pubic arch, and thus the introduction of the catheter is rendered difficult in pregnant women. The pressure caused by the gravid uterus on the vessels in the pelvic cavity necessarily impedes the venous circulation of the surrounding parts; and thus we often see the external organs of

* Maladies des Femmes Grosses, p. 14.

generation, and the inferior extremities, œdematous, and their veins varicose, together with more or less severe pains in those parts, which depend upon compression of the lumbar and sacral nerves.

With most modern authorities, M. Velpeau admits that occasionally the strong articulations of the pelvis are so much relaxed during pregnancy as to allow a certain degree of motion between the bones. This is a point that was long very warmly contested, but in the present day, we believe, few practitioners deny the occasional separation of the pelvic bones during pregnancy. In one respect, however, a discrepancy of opinion still exists. Some regard the softening and relaxation of the pelvic joints as a constant phenomenon of pregnancy, while others regard it as a rare occurrence. Some consider it as a wise precaution of nature to facilitate the act of labour, while others view it as a dangerous disease. We have no doubt upon one part of the subject: we cannot conceive that such a degree of relaxation of the joints of the pelvis, as to lead to any, even the slightest, separation of the bones, could take place without the fact being quite evident, from the greater or lesser degree of difficulty and suffering the patient would feel in attempting to walk, or even to stand; both the difficulty and pain being quite different from that which sometimes arises from the mere weight of the gravid uterus. Now, the existence of these symptoms is very rare in pregnant women; and therefore we infer that the cause of them is absent in the great majority of cases. We believe that a slight softening of the pelvic symphyses generally takes place during pregnancy, but that any degree of motion between the bones is extremely unusual.

In M. Velpeau's account of the general symptoms of pregnancy we find nothing particular to dwell upon. With every other candid writer, he admits that the evidence they convey is far from conclusive. They may all exist in the unimpregnated female; and, on the other hand, pregnancy may go on, and none of them may arise. Still, in most cases, an experienced and careful practitioner will be enabled to form a very strong presumptive opinion.* M. Velpeau particularly insists upon the necessity, in doubtful cases, of an examination by the rectum, and the external examination of the abdomen; two sources of evidence which are in the present day too much neglected. We do not doubt the accuracy of his opinion, that, by external examination, " the existence of preg-

* Upon this subject we would refer the young practitioner to Dr. Gooch's observations, *loc. cit.* p. 198; and to Dr. Montgomery's excellent paper, *loc. cit.*

nancy may be determined in as many women during the second or third months as in the fourth month, by examination per vaginam." But, query, in *how many* women can we resolve the question by either mode of investigation, at either of the periods he mentions. After an examination per vaginam; having in vain tried the "*ballottement*," and when an examination of the abdomen has led to no satisfactory conclusion, auscultation alone remains to resolve the often perplexing problem of the existence or the non-existence of pregnancy; and to this somewhat novel and very interesting topic an elaborate consideration is given.

When Laennec had proved that we might *see* with the ear what was going on in the chest, it was natural to hope that auscultation would soon be applied to other parts of the body, for the purpose of determining the existence of functional derangement or structural disease. MM. Mayor* and Foderé† had already hinted at the subject, in reference to the detection of pregnancy, when M. de Kergaradec‡ declared that utero-gestation might be positively determined by auscultation. Two kinds of "*bruits*" are to be heard in the uterus of a pregnant woman: the one, analogous to, although "plus brusque" and shorter, than that of a feeble respiration, or "*bruit placentaire;*" the other similar to that of the ticking of a watch surrounded by folds of linen, or "*bruit du cœur.*" The first, called by Kergaradec "placental soufflet," is synchronous with the maternal pulsations. M. de K. believes that this soufflet corresponds to the insertion of the placenta, and that it is produced by the passage of the uterine blood into the vessels of the ovum; in most cases it is confessed that a well practised ear is required to detect it at all; and hence many have denied its existence.

M. Velpeau has tried in vain to detect this placental soufflet in very many cases. On the contrary, in others he made it out very distinctly. It was powerful enough in three women, who were delivered at the Hôpital de Perfectionnement, and in two others who served for the "exercises pratiques" at his lectures, for the medical students and female midwifery pupils to hear it very easily and without any previous practice.

We must refer to the work for the detailed account of the conflicting opinions that are maintained respecting the cause of the placental soufflet. The impression upon our own minds, from the few trials we have made, is that the sound

* Bibl. de Geneve, &c. tom. ix. p. 249.

† Dictionnaire des Sciences Medicales.

‡ Auscultation applié à l'Étude de la Grossesse, &c. Paris, 1822.

proceeds from the iliac vessels.' The subject is important, and well worthy further investigations; for, if the present sources of doubt be removed, the existence of the placental soufflet will enable us at once to detect pregnancy, and to determine that the fœtus in utero is living.* This will be a great practical step. The "*bruit du cœur*" cannot be confounded with any other. We may count the fœtal pulsations from 120 to 150 in a minute, whilst the pulse of the mother beats only sixty or seventy in the same space of time. These pulsations are stronger as the fœtus is more developed, and are hardly perceptible until after the fourth month. M. Velpeau has rarely failed to distinguish the action of the fœtal heart when he has been enabled to make a careful examination. We have several times made the attempt, and have only twice succeeded, and in these instances we could distinctly count the fœtal pulse at about 130; the mother's pulse beating seventy-five. In both cases the women were nearly five months advanced in pregnancy. We confess, however, that our ears are not well tuned to stethoscopic sounds, so that others must not be intimidated from our failure in general.

The nature of the "*bruit du cœur*" having been satisfactorily determined, it has not been the subject of controversy; its practical value, however, has been much discussed, and differently estimated. The fair *resumé* appears to be that stated by M. Velpeau. The sound of the heart is a certain sign of pregnancy and of the life of the fœtus. Its power indicates, in general, the vigour and state of health of the infant. Such evidence being of great value in many cases, when accidents occur during parturition, and serious operations are required, and when our practice might be greatly modified by knowing whether the child were living or dead; a very difficult point to determine, as every authority confesses. The simultaneous existence of the beating of the heart at two opposite points of the abdomen would prove that the uterus contained two fœtus; and, if it were distinguished in a case where the uterus was but little developed, a strong suspicion would arise that it was one of extra-uterine fœtation. But the absence of the "*bruit du cœur*," like that of the active or passive movements of the fœtus, is not a demonstrative proof that pregnancy does not exist, or that the child is not alive. This, we doubt not, is the fair and unexaggerated result of what is at present known upon the subject. M. Hohl†

* M. Velpeau refers frequently to Dr. Kennedy's paper "on the Placental Soufflet," of which a full abstract will be found in the Number of the London Medical and Physical Journal for April 1831.—Rev.

† De l'Exploration en Accouchement. 1re Part: Auscultat. Halle, 1833.

would carry our confidence much farther. His opinions are stated by M. Velpeau, with, we think, a very proper doubt as to their solidity.

The chapter on Extra-uterine Fœtation we pass over, with the single remark that, in nearly all such instances, great doubts must exist as to the nature of the case. We have in our possession a preparation of extra-uterine fœtation, (*ovarian*, we believe; M. Velpeau doubts the possibility of such a fact,) at about the third month of gestation. The lady, the wife of a well-known and highly respected physician, did not, of course, want the best medical advice. She died after some weeks' suffering, but the real nature of the case was not discovered until a post-mortem examination was made.

*Fausse Grossesse, (Apparent Pregnancy.)* To all experienced practitioners the fact is well known, that various maladies may give rise to symptoms that very closely simulate pregnancy. We have seen three or four remarkable cases of this kind. Apparent pregnancy may depend upon retention of the menses; irregular menstruation; disease of the uterus, Fallopian tubes, ovaria, or any of the abdominal or pelvic viscera; and, lastly, and most frequently, we believe, from a condition which it is impossible to define, and which comprises cases of imaginary, hysterical, and nervous pregnancy. In the great majority of these cases, an examination *per vaginam* would remove all doubt; but, as M. Velpeau correctly states, such patients are usually so firmly persuaded that they are pregnant, that they will not submit to any examination.

"The treatment of apparent pregnancy must necessarily vary with the cause that gives rise to it; I have merely to observe, that, in hysterical, nervous, or imaginary pregnancy, where there is no appreciable lesion of any part, warm baths frequently repeated are much to be relied on. I must also add, that generally no means we can adopt will succeed in preventing the symptoms simulating pregnancy from running on through the whole period of natural pregnancy, and that before they completely disappear, some of the signs of real labour frequently occur."

We have seen several cases of this fancied pregnancy, but we have yet to be convinced that a mimic labour "frequently" terminates the farce; it does so occasionally, but very rarely. On the other hand, if various maladies give rise to the suspicion of pregnancy, real pregnancy often assumes the appearance of organic disease. Our own and foreign journals contain abundant and many very amusing facts of a chopping boy making his appearance, as much to the annoyance of the deceived doctor as the deceiving damsel. For example,

(quoting M. Velpeau), a woman had been treated for some time for supposed hepatic disease, and her case was made the theme of several elaborate clinical lectures. She was suddenly delivered one morning, much to the astonishment of the profession. The cure was perfect.

The two next questions discussed by the author, of the possibility of determining the sex of the child during pregnancy, and of begetting a male or female at will, we pass over, with but one remark, which is curious, at least. From extensive experiments made by M. Girou* upon animals, it appears that the more vigorous the male is at the moment of sexual intercourse, the greater the chance of the progeny being a male. In the human species it has been said, that, in those countries where polygamy is allowed, as in Persia and Turkey, more girls are born than boys. In Europe, where the laws limit the gentlemen more strictly, the contrary is well known to be the fact. In France the girls are to the boys as 15: 16.

As M. Velpeau has devoted another work† to the Anatomy of the Ovum and Embryology, to which we may give a separate review, we pass over these subjects at present.

From his own experience, as well as from the testimony of others, M. V. is convinced, as we are, that the fact of the occasional protraction of pregnancy beyond the ordinary period is undoubted and incontrovertible. He thinks further, that in the present state of our knowledge, we cannot positively limit the possible duration of utero-gestation; but upon what facts this *unlimited* concession of our deficiency rests, he does not inform us.

The fifth book of the first volume introduces us to the practical subject of *Parturition;* the first chapter is devoted to *Abortion.*

The frequency and causes of abortion are first considered. Among the various causes of abortion one has received more attention from our German and French brethren than from English practitioners, namely, *diseases of the ovum.* Morbid states of the ovum which destroy the embryo are extremely common, especially in the early months of pregnancy.

Since M. Velpeau has devoted a good deal of his attention to the subject of embryology, he has inspected more than two hundred ova before the second or third month of pregnancy: at least half of them were diseased. Such is the inference we

* Revue Médicale, 1828; tom. iv. p. 545.

† Embryologie, ou Ovologie Humaine; fifteen plates. By Alf. A. L. N. Velpeau. 4to. 1834. A splendid and very interesting work.

should draw from our own specimens, as well as from the many preparations contained in our different museums. Among other points connected with the anatomy and physiology of the ovum upon which M. Velpeau particularly insists is the *non*-vascularity of the Chorion and Amnion.* We did not expect therefore to find him assigning as among the ordinary causes of abortion, thickening, opacity, and the formation of adventitious membranes upon the chorion and amnion; for, granting that this is the fact, which we willingly do, the inference is clear that both these membranes *are* vascular.† Morbid Anatomy, in truth, here teaches us that which the extreme delicacy of the parts would render very difficult to demonstrate while they were in a healthy condition. It is of importance that practitioners should bear in mind that uterine hemorrhage is not necessarily followed by abortion. Mauriceau, Boer, Puzos, &c. relate cases of severe hemorrhage, in which utero-gestation still went on to the full period. Under such circumstances abortion is greatly to be apprehended; but it is not inevitable. A knowledge of this fact will modify our practice. In many cases the fœtus dies long before it is expelled by nature from the uterus.

Speaking generally, abortion is more dangerous than labour. Some authors have denied this assertion of Hippocrates: it is however correct. Abortion is a disease. Labour is the termination of a natural function. The most formidable cases of abortion are those in which the ovum is expelled during the existence of some acute disease of the mother.‡ Even if miscarriage takes place when the mother is nearly convalescent from acute disease, her life is in danger. M. Serres states that, in twenty cases of abortion when the women had small-pox, not one of them recovered. Exceptions of course arise; but we know, from practice as well as precept, that they are rare. Our very intelligent friend, Mr. Sweatman, lately attended a woman who was in daily expectation of being "put to bed." She was seized with shivering, fever, &c., and, about twenty-four hours after the occurrence of these symptoms, labour came on, and terminated without any adverse symptom. Two days after, she was covered with confluent smallpox, but she went through the disease very favorably. The child was weak and puny at birth. Variola appeared upon it on the third day, and it died in a

* P. 268, vol. i.

† We have a preparation of an abortion, in which the placenta is diseased. The chorion and amnion are both thickly covered with adventitious membranes firm shreds of coagulable lymph unite the two.—Rev.

‡ Maladies des Femmes Grosses, par Mauriceau, p. 166.

few hours. Our former teacher, Dr. Thynne, whose practical judgment was highly and deservedly respected, used to say, in his lectures, that "if a woman either miscarried or had premature labour from the influence of *any* acute disease, her life was *always* in great jeopardy."

Upon the treatment of abortion, M. Velpeau is very brief. He properly deprecates the indiscriminate use of the lancet.

*On Labour in general.* M. Velpeau considers at some length the essential and necessary causes of labour. It is now admitted that the fœtus is not an active agent at the time of labour. A dead child is expelled with the same facility as a living one. To A. Petit* M. Velpeau assigns the merit of having first proved that the efficient cause of the act of labour is essentially constituted by the contractions of the uterus, and *partly* by contractions of the abdominal and thoracic muscles. An observant practitioner needs no proof of this doctrine; but he who cannot read the book of nature will find a sure guide in M. Velpeau. In one case only can labour be regarded as a partly voluntary act. It is certain that a woman who "bears down," as it is termed, with all her force, who makes the most of her pains, however feeble they may be, will thus accelerate her delivery; and that another may more or less delay delivery, by voluntarily opposing muscular action as much as she can. For example: a woman was admitted for delivery at M. Baudelocque's theatre. Labour went on regularly, and the pupils assembled. The dilatation of the cervix now slackened, and no progress was made during the whole night. The *élèves* were fatigued, and retired. The pains immediately returned, and dilatation again went on. The young men again entered; the phenomena of labour again ceased. Baudelocque, suspecting the cause, gave a hint to the students to retire, but to be at hand, and enter upon a given signal. The patient now began "to bear down," and the head of the child was quickly at the vulva. The spectators were once more brought to the scene of action, and the labour was speedily terminated; for it had now advanced too far to be suspended by any voluntary effort, or moral alarm of the woman. There is nothing at all extraordinary in this fact, but it conveys an important lesson, viz. that every circumstance should be avoided, during the act of labour, that is likely to annoy the patient. Such exceptions, however, do not disturb the general rule, that the will has no other influence upon the process of labour, but through the medium of the diaphragm

* Recueil de Pièces sur les Naissances Tardives, p. 60. Paris, 1766.

and abdominal muscles. M. Velpeau does not venture, however, to declare absolutely, with M. Nægèlé,* that the uterine contractions, accompanied by severe pains, are entirely beyond voluntary control.

We cannot stop to detail the many speculations that have been stated to explain the "*cause determinante*" of labour. M. Velpeau defines it to be "a tendency of the fibres of the body of the uterus to contract upon themselves. The opinions of Levret, Baudelocque, Desormeaux, &c. (p. 436,) upon which M. Velpeau's definition is founded, are physiologically curious, and very plausible.

The process of labour is divided into three periods: precursory signs, dilatation, expulsion. The special phenomena of labour, as uterine contraction, dilatation of the cervix, formation of the bag of waters, and the discharge of mucus, are described minutely, and in a very instructive manner. In "tocology," (to adopt M. V.'s nomenclature,) the word pain is synonymous with uterine contraction. But we must remember that this is but conventional language, employed by practitioners to render themselves intelligible to the lay multitude. Pain and uterine contraction are essentially distinct. It is true that pains are connected with contractions of the uterus; that they arise, progress, decrease, and cease together; and that the energy of the one is usually in a direct ratio to the vehemence of the other: but it is also true that the contrary is sometimes the case. No labour can terminate without uterine contraction, but instances are on record in which the process has been completed without pain.† We never saw a labour entirely without pain, but one lady, well known to the editor, whom we attended four times, had so little suffering, as to utter no expression or sound of complaint whatever, and she declared "that labour was nothing."

*Eutocia,‡ (Natural Labour.)* An average, drawn from numerous private and public sources, leads M. Velpeau to the conclusion "that the active co-operation of the accoucheur is useful about once in fifty times." These calculations are liable to such numerous exceptions, as to be but of little if any real value. Judging from our own experience, we should say that, upon the average, the active "co-operation" of the practitioner is not nearly so often required.

M. Velpeau includes under the term natural labour pre-

* Lehrbuch der Geburtshülfe, 1833, p. 125.

† M. Jourdain, These, No. 214. Paris, 1813.

sentations of the head and face. Six varieties of head presentation are admitted, according to the arrangement of Baudelocque, viz. 1, occiput to the left acetabulum; 2, occiput to the right acetabulum; 3, occiput to the pubis; 4, forehead to the left acetabulum; 5, forehead to the right acetabulum; 6, forehead to the pubis. By Capuron and Maygrier, the third and sixth of these presentations are rejected, and we think with propriety. By all authorities they are admitted to be very rare; by many, they are denied ever to occur.

We would willingly follow M. Velpeau through the minute and very instructive detail he has given of the mechanism of each of these presentations. It far surpasses any description of the mechanism of natural labour which is to be found in any English writer, and the student who will sit down patiently, with a female pelvis and a fœtal head, and then follow the steps of nature so clearly described by the author, will in two hours rise from his task, with more fundamental knowledge of the curious process of labour than he could acquire by mere reading in as many months or years; if ever, indeed, he would thus succeed in obtaining it at all.

Owing principally to the works of Boer and Madame La Chapelle, the doctrine which taught that face presentations were so formidable is now almost exploded, and by the best authorities it is now admitted that, in the majority of cases, such labours are nearly as easy and as natural as if the vertex presented. We perceive, by the by, that some of our London lecturers still remain of the old opinion; but it is clear, if the printed report of their lectures be correct, that they themselves have yet to receive a profitable lesson upon the subject; that, in fact, they mistake altogether the real mechanism of labour in face cases. M. Velpeau will teach them better, or Madame La Chapelle,* if they prefer female instruction.

In the ensuing chapter is described, with equal accuracy and minuteness, "presentations of the pelvis," comprehending the feet, knees, and breech.

We now arrive at the duties of the accoucheur in the lying-in room. That he should be correct in his *diagnosis* is as desirable for his own sake as his patient's. In the opinion of the public, nothing is so easy as to determine immediately whether a woman is in labour or not: the experienced practitioner knows better. That women are themselves frequently deceived, may be easily imagined. But "patients

* Prat. des Accouch. tom. i. 3d Mémoire.—REV.

have frequently been kept for several days confined to bed, even by practitioners, who have at last discovered that several weeks were yet to elapse before the period of pregnancy was complete. A young female, pregnant for the ninth time, felt some pain, and believed she was in labour. Several accoucheurs were called in succession: one felt the membranes protruding; another was quite sure the head was within the cervix; a third was equally certain that the cervix was quite obliterated; a fourth proposed the application of the forceps. On the fifteenth day my opinion was taken, and I found the cervix in the ordinary condition of the seventh month. There was an anterior obliquity of the uterus." M. Velpeau stated that a month would probably elapse before labour came on, and in fact it did not happen for two months. That several accoucheurs could be thus deceived is rather remarkable: but we know, from our own observation, that young practitioners not unfrequently fancy a woman in labour, when she is not, where there is great anterior obliquity of the uterus, and where, of course, the os uteri is thrown very far backwards. They feel the head of the child close to the symphysis pubis, with the uterus thinly expanded over it, and believe the neck is obliterated, when the unchanged os uteri is probably far back towards the sacrum, where they never search for it. By paying due attention to the observations of the author, such mistakes will be easily avoided.

M. Velpeau particularly calls the attention of young practitioners to one circumstance, which is calculated to deceive them, and which, he believes, is not sufficiently known.

"Modern accoucheurs have, with one accord, rejected as apocryphal the recorded cases which appear to prove that labour may commence, that the uterine contractions may be very evident, and that, after having existed for several hours, the labour may be suspended, and delivery not take place *for a month or two* afterwards. Such anomalous examples have usually been cited as proofs of protracted pregnancy, or of superfœtation. It has been supposed that these fruitless efforts marked the natural term of gestation, and that the time that elapsed between their cessation and the occurrence of real labour was in addition to the ordinary term. Now, I am convinced that this imperfect labour, this 'faux travail,' as Levret* called it, is not a chimera. I was called, in March, 1824, to a lady, in Rue d'Orleans: she was in her second pregnancy, and had been suffering for four hours. Her pains were regular, but weak, with long intervals between them. The cervix was soft, and admitted three fingers; it was not com-

* Art des Accouchemens, p. 97.

pletely obliterated. The vertex had began to enter the cervix; and during the pains the membranes were in the vagina, and were smooth and tense; while, on the other hand, I felt the os and body of the uterus harden, and contract with a certain degree of energy. It was now ten o'clock at night. I stated that the labour would not terminate for some hours. I returned home, desiring to be sent for when the membranes ruptured. Not having been requested to attend during the next two days, I presumed some other practitioner had been consulted, and thought no more of the case. Six weeks afterwards I was again sent for, much to my surprise, I confess; for I presumed that delivery had long before taken place. But now the process continued, and the labour was concluded."

M. Velpeau refers to various authorities for nearly similar cases. We do not at all doubt the facts, but we may safely say that, when the process of labour has proceeded so far as mentioned by M. Velpeau, it is *very rarely* suspended so completely, and for so long a period. The suspension of labour for a few hours, without any obvious cause, even when the uterine action has been regular and powerful, is not very uncommon.

For the distinction of the various parts of the fœtus that may present, we refer to the work.

Having established our diagnosis, another question immediately presents itself: how will the labour terminate? Will it be quick or slow? The young practitioner must remember to be very guarded in his prognosis. The duration of labour is so variable, and depends on so many circumstances, which cannot be anticipated, that the most experienced are frequently mistaken in their opinions upon this subject. Nothing but practice can enable a young hand quietly to avoid, and dexterously to parry, the pressing solicitations of his patient to tell her "when it will be over." He must make no positive promises as to duration, but will, of course, console his patient, as soon as he can, by the inspiriting news that "every thing is right:" in other words, that the presentation is natural.

Our readers must look to the work for two or three instructive pages, in which a variety of circumstances are mentioned that will more or less modify the prognosis. We must also omit any formal notice of the general management of the lying-in patient.

We are quite certain that many practitioners give themselves a great deal of unnecessary trouble, and their patients much annoyance, at least, to no purpose whatever, by what is called "supporting the perineum." We give therefore the following short quotation from M. Velpeau.

"The pressure that is employed should be from behind forwards, —from the coccyx towards the pudendum, and not in a lateral direction. The occiput should be made to rise towards the pubis, and not be prevented from descending. It is, besides, only at the moment when the head begins to distend the vulva with some force that we are called upon to interfere at all: before this period the operation can be of no service, and the accoucheur would merely show that he did not comprehend what he was doing."

It is with some hesitation we state our own opinion upon the subject, but the accuracy of it is confirmed by ample and personal experience. *We believe that rupture of the perineum is just as effectually, and more easily, guarded against, by merely preventing the sudden expulsion of the head or shoulders, by pressing upon the part firmly as it descends, with the expanded fingers, as by the ordinary mode of practice.* We doubt the accuracy of Kilian's opinion, that the perineum is in *more* danger from the shoulders than the head of the child.*

Under the head of "Soins relatifs au Fœtus," some judicious hints are given as to when, and how far we are justified in attempting to change the posterior occipital presentations into a more favourable position; the management of face cases, and presentation of the inferior extremities, when assistance is required, are also here mentioned. The treatment of difficult or irregular labour, with a detailed account of the various causes of lingering labour, are next discussed. We must confine ourselves to a few brief comments upon these topics.

M. Velpeau speaks highly of the application of belladonna in cases of spasmodic contractions of the cervix. This remedy was frequently used by La Chapelle, often with benefit, and never with injury, although its use has been proscribed by Kilian.† Upon referring to Kilian, it appears that he infers the same permanent paralytic effect might be produced upon the cervix uteri, as upon the iris, from the belladonna; and that the risk of dangerous hemorrhage, &c. would be incurred. His speculative opinion cannot be admitted, if practical experience supports the favourable opinions of the author, Chaussier, and Conquest,

* Die Schultern sind für den Damm *gewiss bei weitem gefährlicher* als der Kopf, und wir sind fest überzeugt, das die meisten Dammrisse, welche beobachtet werden, beim Hindurchgange der Schultern enstanden sind und vielleicht verhütet worden wären, wenn, was aber gewöhnlich geschieht, die Dammunterstützung bei der Geburt der Schultern nicht vernachlässigt gewesen wäre. Die Operative Geburtshülfe, von H. F. Kilian. Bonn, 1834. Band i. p. 171.—Rev.

† Loc. cit. p. 249.—Rev.

as to the good effects and safety of the treatment. Velpeau says, that the rapidity of its action in dilating the cervix is frequently quite surprising. Such cases are too perplexing to justify us in hastily discarding any addition to our means.

In many works on Midwifery, the practitioner is too unlimitedly restricted from ever rupturing the membranes. The error is on the safe side, we confess. M. Velpeau gives many very good practical hints upon the subject. He has employed the ergot of rye more than thirty times. "In every case," he says, "its action has appeared to me evident and incontestable." Our own experience is more limited: the result of it *exactly the reverse* of M. Velpeau's; but still we doubt not its occasional efficacy, any more than we doubt the utter absurdity of the wild eulogies bestowed upon it by Mr. Mitchell in his essay on the subject. Abdominal compression by means of a bandage, in some cases of lingering labour, is strongly recommended.

*Dystocia,** (Difficult labour.) Hemorrhage and Puerperal Convulsions are the two first subjects treated of under this head. Important as these subjects are, we have only space to say that they are well and practically treated by M. Velpeau; originality can hardly be looked for upon such well-considered themes. He has published a distinct essay on Puerperal Convulsions, which we shall hereafter notice; but we must just advert to one error he has committed, especially as the authority of Dr. Merriman, upon all obstetrical subjects, must greatly influence the opinions of others. M. Velpeau states (p. 121, vol. 2,) that forty-eight cases of convulsions occurred to Dr. Merriman in two thousand labours; whereas the fact is, that in 2947 cases, attended by Dr. M. himself, only five were complicated with convulsions. The remaining forty-three cases mentioned by Dr. Merriman† were *consultation* cases, and consequently no average of the ordinary frequency of the disease could be drawn from them. Upon the same subject also M. Velpeau must, *we presume*, have fallen into a similar mistake with respect to M. Gehler, whose statement is thus quoted. "Gehler‡ dit que, sur plus de 300 accouchemens laborieux, *il n'en a vu que 22* cas" of convulsions. In upwards of 23 years we have ourselves attended more than 2000 cases of labour, and a great majority of them in low life. Three of these cases only were compli-

† On Difficult Parturition, p. 147. See also a letter, in correction of M. Velpeau's mistake, from Dr. Merriman: Edinburgh Medical and Surgical Journal, January 1835, p. 240.—REV.

‡ Bibl. de Chir. du Nord, tom. i. p. 346, 347.

cated with convulsions. The women all recovered. The children were born dead. The following subjects are treated of in succession as causes of accidental or essential Dystocia: Prolapsus of the Funis; Shortness of the Funis; Aneurism; Asthma; Hydrothorax, &c.; Hernia; Syncope; Ruptures of the Uterus; Tumours in the Pelvis; Calculi in the Bladder; Malformation of the Vagina; Tumours of the Vulva; Inversion of the Vagina; Disease of the Cervix; Displacement of the Uterus; Obliquities of the Uterus. We must offer a comment or two upon the latter subject.

The anterior obliquity of the uterus is perhaps the only one that requires any particular attention during labour. We have already referred to it as a not unfrequent source of error to the young practitioner. M. Velpeau states that, whenever he has met with this very common deviation from the ordinary position of the uterus, the labour at first makes but little progress, but subsequently becomes powerful, and requires no particular assistance.

"The most distinguished writers, and Baudelocque himself, have advised us, for the purpose of remedying this inconvenience, to hook one or two fingers into the os uteri, and to depress the cervix towards the centre of the pelvis, between the pains, and to retain it in that position during the contractions; or to perform the Cæsarian operation through the vagina, the only means of preventing gangrene or rupture of the uterus, &c. Judging from my own experience, I am inclined to think with Smellie,* who maintains that the deviations of the cervix backwards require neither turning nor artificial dilatations, recommended by Deventer; that, in such cases, artificial means are rarely necessary; and that, in more than one instance, the practitioner has injured either the mother or child by his impatience. For a long time I was led by the doctrines of the books. I drew upon the cervix, and did all I could to bring it into the centre of the excavation. I succeeded, it is true; but very frequently remained many hours with the patient. In one case I was obliged to have a substitute, a pupil, who neglected the instructions I had given him. After an absence of three hours, I found the cervix completely dilated, the membranes ruptured, and the head low down in the pelvis. From this time I have done nothing in such cases, and nature has always restored the parts to a natural position."

M. Velpeau does not assert that nature never requires to be assisted in these cases of anterior obliquity of the uterus, but that the exceptions are few in which the practitioner is justified in interfering. We are convinced that M. V. takes a correct view of the subject, and we cannot refrain from de-

* Midwifery, vol. i.

precating the advice of Dewees* and others to introduce the hand, hook the os uteri upon the finger, and draw it towards the centre of the inferior strait, &c. We, like M. Velpeau, have also given several fair trials to this mode of practice, and also, like him, our experience has taught us that it is unnecessary in the great majority of the cases referred to. It is obvious that if the practitioner *always* has recourse to the manual interference advised by Dewees, in cases of anterior obliquity of the uterus, he never can positively determine how much he has done, or how much *nature* herself has done in hastening and terminating delivery. Let him then trust to nature in a few such cases, or, at least, be satisfied with placing the patient on her back, and supporting the fundus uteri with a towel, and she will teach him the same lesson that she has taught M. Velpeau and ourselves, that she generally requires no aid.

Labour may also be rendered difficult from causes connected with the fœtus: its volume may be excessive, from malformation, monstrosity, or disease; and these subjects are considered at some length by the author. Preternatural presentations are often also the cause of difficult labour.

M. Velpeau doubts that the *trunk* of the fœtal body ever presents in a transverse position at the brim of the pelvis, either before or after the rupture of the membranes, if the child is at the full period and full grown. "That such positions may exist, the horizontal diameter of the uterus must be greater than the perpendicular diameter; and, granting that this can be the case before the beginning of labour, can we conceive that such a disposition could be maintained during the contractions of the uterus?" Mauriceau, Deventer, Smellie, &c. have given plates of such presentations, but none of them state they are taken from nature; and M. Velpeau believes "qu'ils sont tous d'imagination." We believe he is right, from our own observation, as well as from the immense number of labours that have been reported by foreign and English authorities, in which no single instance of such presentations occurred; *e. g.* La Chapelle mentions 40,000; Merriman, 20,000; Riecke, 220,000! &c. &c.

After having given his opinions of the various modes in which an evolution of the child, or a change in its position, is sometimes spontaneously effected, in utero, M. Velpeau refers to many authorities, and briefly sketches from them 137 cases of the kind; 125 of the children were born dead. The following are his practical conclusions as to presentations of

* System of Midwifery, p. 89 et seq.—REV.

the trunk: 1st. That all positions of the trunk, which cannot properly be referred to presentations of the shoulder, back, or the anterior part of the thorax, may be classed as inclined positions of the breech or head. 2d. That presentations of the shoulder are, in fact, the only ones which demand particular attention, inasmuch as nature converts all others into shoulder presentations. 3d. That the child is never placed in the uterus in a really transverse position, and that we may occasionally expect that the most disadvantageous positions will be spontaneously converted into favourable ones. 4. That there are cases, and numerous ones too, in which artificial assistance is not necessary, although the fœtus presents by neither extremity of its long diameter.

We believe these remarks are substantially correct, and they are valuable to the student, inasmuch as they relieve him from the necessity of studying a variety of different positions of the trunk, which he will find in books, and which he *may make* with his leathern fœtus and phantom, as those who have described them have probably done, but which he will not find in practice.*

Occasional exceptions to M. Velpeau's dicta, it is true, may arise; for nature, as we all know, refuses to be confined by any of our abstract and scholastic arrangements.

"The *indications* to be fulfilled in unfavorable positions necessarily vary, according to a variety of circumstances. If the liq. amnii has not escaped, there is nothing to do; we must wait for the dilatation of the cervix. If there is obliquity of the uterus, we must try to restore it to its natural position. If the head projects over the brim of the pelvis, we must endeavour to press it into the inlet. When the fœtus is so moveable, that the head, the shoulder, or some other part, presents alternately at the orifice, it is proper to rupture the membranes, without waiting long, at the moment we are certain the head is presenting at the inlet. But, if the cervix is sufficiently dilated, if the membranes have ruptured, or are on the point of rupturing, it is necessary to determine, without delay, if it be advisable or not to introduce the hand into the uterus. We may dispense with this operation, says Denman, in numerous cases, inasmuch as the uterus will often produce *spontaneous evolution;* and, if the child presents double, its expulsion would not even then be quite impossible. In every case, French accoucheurs contend, on the contrary, that we should act immediately; for they

* "Man schrieb nehmlich damals und grossentheils thut man es noch jetzt, so viele falschen Kindeslagen in ein Handbuch auf, als der Autor in dem vor ihm stehenden Phantome nur immer machen konnte. Solche *Phantom-kindeslagen* sind aber keine *Naturkindeslagen,* wie sie uns das lebende Weib zur Beobachtung darbietet!" Kilian, 363. Die Operative Geburtshülfe. Bonn, 1834.—Rev.

argue, that the longer we wait, the more the uterus contracts, and the more difficult it is to introduce the hand, and perform the operation of turning." (P. 275.)

M. Velpeau is quite in error as to Dr. Denman's opinion upon the subject of Spontaneous Evolution. The first communications upon this point which Denman published were two papers in 1785.* In the first he says, "with respect to the benefit we in practice may derive from the knowledge of the fact, (spontaneous evolution,) I may be permitted to observe, that the custom of turning and delivery by the feet in presentations of the arm will remain *necessary and proper in all cases in which the operation can be performed with safety to the mother.*" In the second paper Dr. D. says,† "The fact of spontaneous evolution being ascertained, I must leave it to other practitioners to determine upon the particular cases in which this event may be reasonably expected and properly waited for." In the first edition of his Midwifery published in 1795, and in the seventh edition published by Dr. Waller, Dr. Denman again says, "Yet the knowledge of this fact (spontaneous evolution,) however unquestionably proved, does not free us from the necessity and propriety of turning children presenting with the superior extremities, *in every case* in which that operation can be performed with safety to the mother, or give us a better chance of saving the child." Further, Dr. Denman says, and truly too, that the knowledge of the occasional occurrence of spontaneous evolution will be a relief to the practitioner's mind, and prevent him from having recourse to hazardous operations, in those cases where turning either cannot be effected without great danger to the patient, or where it is altogether impracticable.

Now in all this we see the caution which characterized every statement of our venerated authority—Denman. No man was less likely than he was to fall into the mischievous error of jumping to hasty conclusions, from imperfect premises. Dr. Walshman declared at the Medical Society of London,‡ "that Denman had done more harm by his article on Spontaneous Evolution, than he had done good by all the rest of his writings." In defence of DENMAN we will no further retaliate upon Dr. Walshman, than by asserting that, whatever arose of "harm" from his papers on Spontaneous Evolution, must be attributed to the blunderers who mistook his meaning, or to those vacillating practitioners

* London Medical Journal, vol. v. By Symons.—REV. † Ibid.

‡ Lancet, 1827, vol. ii., p. 88.

who, glad to have some excuse for escaping from an operation which they shrunk at, chose to do nothing, when attention to Denman would have taught them to act with promptitude.

We presume, for he gives no reference to any work of Dr. Denman's, that M. Velpeau quotes him at second-hand, and it is passing strange to us that many of our own writers have fallen into the same mistake. Dr. Blundell, for example,* says, "Observing these 'spontaneous evolutions,' as he significantly called them, and unwilling to interfere without need, Dr. Denman advised that, in arm presentations, we should *always!* confide the delivery to the natural efforts, abstaining from the introduction of the hand into the uterus." Dr. Gooch is reported to say† that Denman published his cases of spontaneous evolution, "together with the inference that arm presentations may be *entirely!* left to nature: this conclusion was, however, invalidated by his subsequent experience." How these, *we are sure, involuntary* misstatements of Dr. Denman's opinions can have arisen, we are at a loss to determine. M. Velpeau appears, too, to assume that the practice of turning, as soon as we can *with safety* to the mother, is peculiar to the French school. He is quite mistaken: the doctrine he advocates is taught in every English school, and acted upon by every well-informed English practitioner; and by a recent and very able German writer, we also find it insisted upon.‡

For the detailed consideration of the various circumstances which demand, and the mode of performing the operation of, turning we must refer to the work. M. Velpeau is one of the few modern authorities who contends for the propriety of frequently endeavouring to convert preternatural presentations into presentations of the head by "*la version céphalique.*"

We must now be very brief in our arguments and extracts. Our opinion is that this operation must always be very difficult, frequently impossible, and rarely justifiable.§ We fully

* The Principles and Practice of Obstetricy, p. 383; edited by Dr. Castle.

† Practical Compendium of Midwifery, p. 238: from Dr. Gooch's Lectures. By George Skinner.

‡ "Nicht umhin aber können wir, unsere weniger erfahrenen Geburtshelfer alles Ernstes darauf aufmerksam zu machen, dass es zu den unverantwortlichsten Thorheiten gehören würde, wollte irgend einer unter ihnen in einem schwierigen Falle als müssiger Zuschauer auf eine *Selbstwendung* des Kindes warten und darüber die unschätzbare Zeit und den rechten Augenblick zur Wendung selbst versäumen." Die Operative Geburtshülfe, band i. 365. Von Dr. H. F. Kilian. Bonn, 1834.—Rev.

§ Vide Pratique des Accouchemens, par Madame La Chapelle; tom. i. p. 79. —"The chance of success in this way is very trifling." Merriman on Difficult Parturition, fourth Edition, p. 82.—Rev.

agree with Burns,* who says, in reference to the old practice of endeavouring to remove the presentation, and to bring the head to the os uteri, (which, by the way, was followed from the time of Hippocrates to Ambrose Paré,) " there may however be cases, where it would not only be safe, but also more proper, to resort to the old practice, although as a general rule it ought to be abandoned. For example, it sometimes happens that when the hand or shoulder presents, the head rests on the edge of the brim of the pelvis, and, if we return the presenting part, the uterus is so stimulated to a vigorous action, by the introduction of the hand, that the head is thrown off the brim of the pelvis, and descends as in a natural presentation.† Dr. Gooch thus succeeded many times in converting an arm into a head presentation. Three cases of this kind have occurred to ourselves. But here the interference does not deserve the title of "*version céphalique*." We find from M. Velpeau himself, that his countrymen coincide with us. " In spite of what some moderns have said, nobody at Paris has thought of putting in practice *la version céphalique*. Madame La Chapelle goes so far as to deny the possibility of the operation," p. 289. Joerg, of Leipzig, is a great authority, and therefore we are tenacious of what he says. M. Velpeau thus quotes his opinion from Burns.‡ " M. Joerg n'a pas craint *de poser en regle* que dans la version, il faut ramener la tête, &c." p. 291. Now the expression used by Burns to convey Joerg's opinion is " that he *admits the propriety* of bringing down the head, &c." We are not hypercritical, we hope, when we submit to M. Velpeau that he has translated the passage too strongly on his side of the question. Barely " to admit the propriety" of a practice is not equivalent to "laying it down as a rule." Wigand states that the head may often be placed at the brim of the pelvis, when the presentation is preternatural, without introducing the hand at all; by acting upon the uterus through the abdominal parietes, and placing the patient at the same time in an appropriate position. Velpeau has twice adopted this plan with success *before the rupture of the membranes.*

The chapter on the Forceps is short, but rich in practical instruction. M. Velpeau confines the use of the instrument to the head, in common with most modern authorities. He employs Levret's instrument, with a slight modification. Of the straight forceps, which we hold to be preferable to the curved in the great majority of cases, especially for young

* Principles of Midwifery, p. 384, eighth Edition, 1832.

† Vide Gooch's Compendium of Midwifery, p. 237.

‡ Midwifery, p. 384, (note;) eighth Edition.

practitioners, little or nothing is said: it is rarely, if ever, we believe, used in France. Notwithstanding what M. Velpeau says, we remain extremely sceptical (we must not use a stronger term,) as to the ease or safety with which the forceps can be applied *above the brim of the pelvis;* and we strongly urge the inexperienced practitioner, at least, not to trifle with his patient's life, by attempting to use the instrument in such cases. We oppose to M. Velpeau's opinions those of a distinguished French professor, Capuron,* who says, "Nous sommes presque tentés de douter qu'on ait jamais réussi, à moins que la tête n'eût commencé de s'engager;" and Madame La Chapelle's, who says, "Mais lorsque rien n'est engagé dans l'excavation l'application devient *très difficile* et souvent dangereuse."† Kilian also thus condemns the practice,‡ "Above all, the accoucheur must regard it as his strict duty not to think of the use of the forceps, until the head is firmly fixed in the brim of the pelvis." M. Velpeau may think Kilian's note a little uncharitable. We give it§ without meaning to insinuate, in the most distant manner, that *he* need wince at the German professor's severity; and with the free confession that it will apply in England as well as in France. Burns says,‖ "When it (the head,) is altogether above the brim, or only a small part, after many pains, has entered, the forceps cannot be used." Blundell's name is frequently quoted: it deservedly carries weight; but he speaks very indefinitely upon the subject. Thus,¶ "The cases, of all others the most frequently requiring the long forceps, consist of those laborious labours in which the child's head is detained *at* the brim of the pelvis."

We feel deeply interested upon this subject, and regret that we cannot more fully discuss it. We vouch for the truth of the following statement, although we are not at liberty to mention names. A practitioner of distinction and great experience employed the long forceps in this country in twelve cases. In some the head was above the brim; in some it had

* Cours d'Accouchemens, p. 561.—Rev.

† Op. cit. tom. i. p. 73.—Rev.

‡ Op. cit. p. 607.—Rev.

§ "We cannot conquer the belief that, in a number of the cases, particularly of the practitioners in France, in which the forceps have been said to be applied above the brim of the pelvis, there has been some delusion, (auf Täuschung beruht;) for it is not every applier of the forceps who can determine whether the head is within or above the brim of the pelvis. Practitioners have there very commonly, partly from error, and partly from vanity, represented cases in which the head was high up in the pelvis, to be instances of the head above the brim." Operative Geburtshülfe, p. 607.—Rev.

‖ Midwifery, eighth Edition, p. 451.—Rev.

¶ Op. cit. p. 502.—Rev.

partly entered. In every case the child was born dead. Four of the women died, and every one of them suffered severely; some from inflammation, others from laceration of the soft parts. On the other hand, it is certain that the ordinary doctrine of English teachers and English writers, of confining the application of the forceps to cases where the head is low enough in the pelvis for the ear to be felt, limits the use of the instrument within too narrow bounds; but we think that a young practitioner should confine himself for some time to this rule, although we deprecate it as the guide for him whom experience has rendered dexterous.

The use of the Lever; Artificial Labour; Division of the Symphysis Pubis; the Cæsarian operation; Craniotomy; and the Natural Phenomena which follow the Expulsion of the Fœtus, are the next subjects considered; but of which we can only enumerate the titles.

The work concludes with a brief sketch of the principal diseases to which newborn children and lying-in women are liable.

Many pages of interesting matter we thus pass over, but once more we must plead "our limits," and close our notice of a work which we have perused with much attention, as a duty to our readers; and with much attention also, from the great pleasure and information we have derived from it ourselves. It is indeed a model of industry, and must be of immense value to every one who wishes to become acquainted with the erudition and the practice of midwifery, from the earliest times up to the present day. We cannot conscientiously say it is a book we should put into the hand of a tyro in obstetrical studies. It is above him; it is too profuse in references to various authorities, ancient as well as modern, for his purpose, who requires rather a succinct statement of practical facts, than an elaborate survey of all that has been said. In making this remark, we are far from meaning to undervalue the utility of the labour M. Velpeau has bestowed in informing us of the errors of our predecessors, as well as the improvements of our contemporaries. To know even the errors of those who have preceded us is by no means a useless acquisition: they should be remembered, as the pilot bears in mind the shipwrecks of those who have navigated before him, that he may himself steer a safer course. That other editions of M. Velpeau's work will be called for, we have no doubt; and we beg to suggest, that in one respect there is room for improvement: not only the names of the authors referred to should be given, but the editions and pages of the works which are quoted should be stated.

The plates are very neatly executed, and the subjects they depict physiologically interesting and practically useful: they comprise illustrations of the anatomy of the pelvis and female organs: anatomy of the ovum; various positions of the fœtus; application of instruments, &c.

M. Velpeau will, we are sure, forgive us if we indulge in a smile at one of his arguments. In speaking of the period when the embryo first becomes visible in the uterus, (the mystery which so many have in vain endeavoured to unravel,) he rejects as fabulous or fanciful the well-known case of Sir Everard Home and M. Bauer,* which, to his astonishment, created a great sensation in the scientific world of London. We admit his doubts; but then we doubt, in turn, the value of the fact he himself adduces to fill up the physiological hiatus he laments.

M. Velpeau has had an opportunity of examining three early ova. He considers "†the third as the most precious that has yet been met with. It compels us to admit that the embryo exists in ovula of ten days, and perhaps before: here is the history. A sage-femme, who attended my lectures, was at the last day of her menstrual discharge, when her husband returned from Rouen, where he had sojourned for six weeks. *Les approches conjugales* did not take place till the following day, and on the thirteenth day the lady, who was already the mother of six children, miscarried. The ovulum, which she immediately sent to me, might indeed be less, but could not, as we perceive, be more than twelve days old. It is the only case within my knowledge in which the age of the ovulum can be so positively determined. The embryo was here very distinct, as well as the vesicles and all the membranes. The funis itself already existed, and the form of the little embryonic body left no room for mistake."

Now we really perceive no compulsion of belief in this case. It is evident that the lady had more physiological zeal than female delicacy, who could instruct M. Velpeau as to the exact time "les approches conjugales eurent lieu," &c. Every *sage femme* is not necessarily a *femme sage*, and it is unquestionably possible that the ovulum might have been more than twelve days old, in spite of the flow of the catamenia immediately preceding. We are much mistaken if M. Velpeau himself, upon reflection, will not agree with us, that

* Philosophical Transactions, 1817, p. 252.

† We quote from M. Velpeau's "Embryologie," p. 51, Brussells edition. The "cas précieux" is more briefly mentioned in the Traité des Accouchemens. —Rev.

a *still more* precious case must be placed on record, before we can be satisfied

> "How the dim speck of entity began
> T'extend its recent form, and stretch to man."

